# Rapid Recognition of Carfilzomib‐Induced Thrombotic Microangiopathy and Early Application of Eculizumab Successfully Rescued Two Relapsed Myeloma Patients

**DOI:** 10.1002/cdt3.70036

**Published:** 2026-02-15

**Authors:** Zi Wang, Hua Zheng, Wei Wang, Xiaoxiao Shi, Yunpeng Wei, Chao Li, Junling Zhuang

**Affiliations:** ^1^ Department of Hematology Peking Union Medical College Hospital Beijing China; ^2^ Department of Nephrology Peking Union Medical College Hospital Beijing China; ^3^ Department of Internal Medicine Peking Union Medical College Hospital Beijing China

Carfilzomib (CFZ) is an irreversible proteasome inhibitor currently approved for the treatment of relapsed multiple myeloma (MM). It has been implicated as a cause of thrombotic microangiopathy (TMA) and mostly occurs after two courses of chemotherapy. The incidence, risk factors, and treatment of CFZ‐related TMA remain unclear. Here we describe two cases.

Carfilzomib (CFZ) is an irreversible proteasome inhibitor currently approved for the treatment of relapsed multiple myeloma (MM). It has been implicated as a cause of thrombotic microangiopathy (TMA) and mostly occurred after two courses of chemotherapy. The incidence, risk factors, and treatment of CFZ‐related TMA remain unclear. Here we describe two cases.

Case 1 was a 70‐year‐old woman. She was diagnosed with MM in 2010, λ light chain with Durie–Salmon Stage III, international staging system (ISS)‐stage I. kidney function was normal at baseline. Complete response was achieved after bortezomib‐based front‐line regimen (bortezomib, epirubicin, and dexamethasone, PAD) and thalidomide maintenance therapy. The first relapse occurred after 5 years, when she received thyroidectomy for papillocarcinoma. Subsequently, she underwent multiple courses of chemotherapy incorporating regimens containing bortezomib, lenalidomide and ixazomib, yielding unsatisfactory disease control. The 24‐h urine λ levels fluctuated between 1000 and 2500 mg, and serum FLC‐λ ranged from 350 to 500 mg/L. In pursuit of improved outcome, she was treated with a KPD regimen consisting of carfilzomib (30 mg intravenously on Days 1 and 2), pomalidomide (4 mg orally on Days 1–21), and prednisone (40 mg orally once weekly), at the age of 70 years in December 2023. Complete blood count (CBC) was in the normal range.

On the second day after first dose of CFZ and pomalidomide, she abruptly developed fatigue, dark urine, reduced urine output and fever with a peak body temperature of 38.5°C. Additionally, she experienced bilateral lower back pain and reduced urine output. She presented to the emergency room on Day 4 of chemotherapy, also exhibiting abdominal symptoms, notably nausea, vomiting, and diarrhea. Laboratory examinations revealed hemoglobin (Hb) level of 63 g/L, platelet (PLT) count of 8 × 10^9^/L, a significant presence of schizocytes in the peripheral blood smear, and a lactate dehydrogenase (LDH) level of 2499 U/L. The Direct Antiglobulin Test (DAT) yielded negative results. Percentage of reticulocyte was 7.3%. Serum free hemoglobin concentration was 39.6 mg/dl (reference range: 0–5 mg/dL). Her serum creatinine (SCr) sharply elevated from 60 to 510 μmol/L, and blood urea nitrogen (BUN) surged from 5.96 to 34.51 mmol/L. Urinalysis showed microscopic hematuria (200 cells/μL) and proteinuria increased from 275 mg/L before treatment to 1040 mg/L after treatment.

The patient exhibited prominent features of microangiopathic hemolytic anemia (MAHA) and acute kidney injury (AKI). Immediate single membrane plasma exchange was initiated in first‐aid room. Supportive therapy commenced with thrombopoietin (TPO), erythropoietin (EPO), folate, and vitamin B12 supplementation. She was admitted to nephrology ward on Day 5 after 7 plasma exchanges, exchanging 2.0–3.0 L plasma daily. Although PLT count improved from 8 × 10^9^/L to 77 × 10^9^/L, LDH decreased from 2499 U/L to 328 U/L, the patient's hemoglobin level dropped from 63 g/L to 49 g/L and SCr increased from 524 μmol/L to 599 μmol/L. Peripheral blood smear still exhibited a significant number of schizocytes.

Following multidisciplinary consultations, abnormal activation of the alternative complement pathway was considered as a potential cause of TMA in this patient. Her ADAMTS13 activity was assessed at 54%. Complement C3 levels were within the reference range (0.76–0.93 g/L), whereas complement C4 decreased to a nadir of 0.05 g/L. Factor H (FH) and its autoantibodies were both within normal limits. Markedly elevated C5b‐9 levels (748 ng/mL; reference range: 75–219 ng/mL) and CH50 of 32.4 U/mL (reference range: 23.0–46.0 U/mL) supported complement pathway involvement. We evaluated the patient's complement pathway‐associated genes, including *CD46, CFB, CFH, CFHR1, CFHR3, CFHR4, CFI, C3*, and *THBD*; all results yielded negative findings.

On Day 14 after chemotherapy, the first dose of intravenous eculizumab 900 mg was administrated. At 1 week post‐infusion, the patient's hemoglobin improved from 49 g/L to 73 g/L. SCr recovered from 598 μmol/L to 262 μmol/L, BUN decreased from 28.11 mmol/L to 21.21 mmol/L. C5b‐9 level decreased from 748 ng/mL to 270 ng/mL, and CH50 from 32.4 U/mL to 19.5 U/mL. Following two doses of eculizumab infusion, the patient was discharged with hemoglobin 76 g/L, PLT 423 × 10^9^/L, LDH 387 U/L. SCr 174 μmol/L, and BUN 16.37 mmol/L. During hospitalization, intravenous ceftriaxone was administered as prophylaxis against meningococcal infection due to the inaccessibility of meningococcal vaccines (antibiotics were recommended to initiate following eculizumab administration to mitigate the risk of meningococcal infection until vaccination could be completed).

During post‐discharge follow‐up, the patient's hemoglobin, SCr, and BUN levels remained stable. Two weeks after the second dose of eculizumab, the PLT count decreased from 405 × 10^9^/L to 44 × 10^9^/L, while LDH increased from 328 U/L to 394 U/L. Subsequently, a prompt rise of PLT from 50 × 10^9^/L to 162 × 10^9^/L was achieved after the third dose of eculizumab at 900 mg, meanwhile pneumococcal vaccine was injected on Jan 2. She was on regular eculizumab injections for a total of 3 months. No recurrence of TMA was observed (Figure 1).

**Figure 1:**
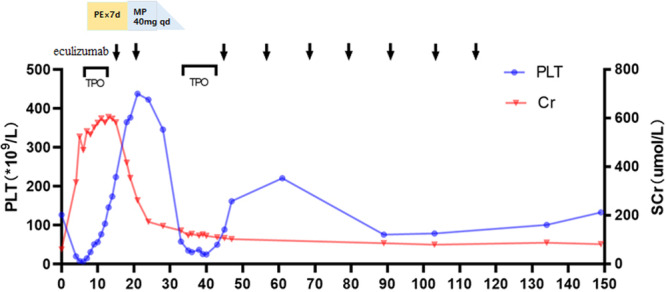
The treatment process of Case 1 and the trend chart of changes in PLT and SCr over time. (The horizontal axis represents the number of days since the diagnosis of TMA. The blue curve represents the level of PLT variation. The red curve represents the level of SCr variation. The black arrow represents the time point of injection of eculizumab. The black box represents the time window for TPO injection). MP, methylprednisolone; PE, plasma exchange; PLT, platelet; SCr, serum creatinine; TPO, thrombopoietin.

Case 2 was a 63‐year‐old woman. She presented with numbness in the fingertips and was diagnosed with MM on 2018, IgGλ, ISS Stage I, Durie‐Salmon Stage IA. She received five cycles of BCD regimen (bortezomib, cyclophosphamide, dexamethasone) and five cycles of BRD regimen (bortezomib, lenalidomide, dexamethasone), achieving a partial response (last follow‐up in April 2020). On June 15, 2020, autologous hematopoietic stem cell transplantation (ASCT) was performed followed by dual drug maintenance with IR (ixazomib and lenalidomide). Due to adverse effects of ixazomib, single medication of lenalidomide was ongoing since November 12, 2022. From February 2023 to November 2023, due to biochemical disease relapse, a total of eight cycles of DRd regimen (daratumumab, lenalidomide, dexamethasone) were administrated as the third line treatment. However, gingival plasmacytoma developed as progressive disease in October 2023.

From November 2023 to January 2024, she was treated with three cycles of KD chemotherapy (carfilzomib and dexamethasone). The response was stable disease. Her last medication was given on January 24, 2024, with carfilzomib (98 mg intravenously) and dexamethasone (20 mg orally). Subsequently, she developed cough, hemoptysis, and dyspnea. In the emergency room, nucleic acid of influenza virus B was positive. Oseltamivir was prescribed. Hemoptysis worsened on January 25, accompanied by wheezing and shortness of breath. Laboratory tests demonstrated grade 4 thrombocytopenia with a platelet count of 12 × 10^9^/L to 1 × 10^9^/L, along with a hemoglobin level of 90 g/L and progressive renal impairment (serum creatinine increased from 53 μmol/L to 185 μmol/L). Additionally, proteinuria was ≥ 3.0 g/L, significantly higher than the baseline level of 0–0.3 g/L before treatment. The presence of schistocytes in the peripheral blood smear, an elevated reticulocyte percentage (2.68%), LDH level of 1150 U/L, and free hemoglobin concentration of 8.1 mg/dL (reference range: 0–5.0 mg/dL) confirmed the diagnosis of TMA. Due to respiratory failure associated with hemoptysis, the patient was transferred to the intensive care unit (ICU) and intubated.

Overactivation of the complement alternative pathway was considered as a potential cause of TMA in this patient. Her ADAMTS13 activity was measured to be 57.36%. Complement C3 levels decreased from 1.81 g/L to 0.64 g/L (normal range: 0.76–0.93 g/L), as well as complement C4 reaching a minimum of 0.08 g/L. Other tests included FH at 231.05 ng/mL (reference range: 246.60–417.69 ng/mL) and Factor I at 10 ng/mL (reference range: 12.26–333.02 ng/mL). Stool routine test and bacteria culture were unremarkable. It was considered that the patient may had a potential deficiency in the complement alternative pathway inhibitors, leading to complement‐mediated TMA.

Plasma exchange was initiated on January 27, 2024, with daily 2000 mL for 6 days. Since the vital signs were stable, the patient was transferred to hematology ward on February 1 and administrated with eculizumab 900 mg. One week later, her hemoglobin improved from 53 g/L to 70 g/L. SCr decreased from 227 μmol/L to 123 μmol/L, and BUN decreased from 25.63 mmol/L to 11.72 mmol/L.

After 2 weekly doses of eculizumab, the patient was discharged with a hemoglobin level of 70 g/L, PLT count of 52 × 10^9^/L, and LDH level of 439 U/L. Starting from January 28, moxifloxacin (0.4 g once daily) was administered prophylactically against bacterial infection. This measure was necessitated by the inability to access meningococcal vaccines, as antibiotic prophylaxis is recommended for meningococcal infection risk mitigation during eculizumab therapy until vaccination can be completed. One month after the second dose of eculizumab, the patient's PLT count recovered to 213 × 10^9^/L, and LDH decreased to 278 U/L. Until the last follow‐up, her TMA did not recure (Figure 2).

**Figure 2:**
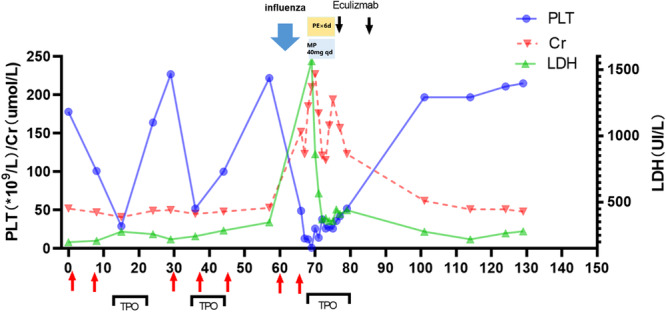
The treatment process of Case 2 and the trend chart of changes in platelet count and serum creatinine levels over time. (The horizontal axis represents the number of days since the diagnosis of TMA. The blue curve represents the level of PLT variation. The red curve represents the level of SCr variation. The green curve represents the level of LDH variation. The black arrow represents the time point of injection of eculizumab. The black box represents the time window for TPO injection). LDH, lactic dehydrogenase; MP, methylprednisolone; PE, plasma exchange; PLT, platelet; SCr, serum creatinine; TPO, thrombopoietin.

We reviewed and summarized case reports of CFZ‐induced TMA (CFZ‐TMA) published from 2014 to 2025. Cases with complete data on treatment modalities, duration, and outcomes were collected, and the outcomes of each therapeutic approach were summarized (detailed in Table 1). As shown in Table 1, a total of 32 cases met the inclusion criteria and were included in the analysis. Among these, four cases received supportive therapy: three of the four patients achieved normalization of renal function within 4–8 weeks post‐treatment, while one patient had persistent renal dysfunction without recovery. Eight cases were treated with therapeutic plasma exchange (TPE) alone: seven of these eight patients showed recovery of renal function to normal within 1 week to 9 months post‐treatment, and one patient had persistent renal dysfunction without recovery. Nineteen cases received eculizumab therapy: 15 of the 19 patients achieved normalization of renal function within 2–12 weeks post‐treatment, while the remaining 4 patients had persistent renal dysfunction without recovery.

**Table 1:** Carfilzomib‐induced thrombotic microangiopathy: Treatment and outcomes.

Author‐year	Number of cases	Gender	Age (year)	Therapy	Time from treatment ‐ renal function return to normal
Hobeika 2014 [1]	1	M	62	Supportive care	8 weeks
Sullivan 2015 [2]	1	M	74	TPE alone	1 weeks
Lodhi 2015 [3]	1	M	63	TPE alone	7 weeks
Qaqish 2016 [4]	2	M	73	TPE alone	5 weeks
		F	72	TPE alone	9 months
Chen 2016 [5]	3	F	66	Supportive care	4 weeks
		M	63	Supportive care	8 weeks
		M	58	Supportive care	Not achieved
Gosain 2017 [6]	1	F	61	Eculizumab	6 weeks
Portuguese 2018 [7]	2	M	59	Eculizumab	4 weeks
		M	66	Eculizumab	Not achieved
Moliz 2019 [8]	1	F	71	Eculizumab	4 weeks
Bhutani 2020 [9]	1	F	44	Eculizumab	7 weeks
Casiez 2020 [10]	1	M	66	Eculizumab	8 weeks
Blasco 2021 [11]	4	F	59	TPE alone	4 weeks
		F	75	TPE alone	4 weeks
		M	60	TPE alone	Not achieved
		M	41	Eculizumab	4 weeks
Rassner 2021 [12]	2	M	43	Eculizumab	8 weeks
		M	59	Eculizumab	6 weeks
Freyer 2021 [13]	1	F	51	Eculizumab	Not achieved
Eigbire‐Molen 2022 [14]	1	F	62	Eculizumab	2 weeks
Scheggi 2022 [15]	1	M	70	Eculizumab	6 weeks
Catanese 2023 [16]	1	M	50	Eculizumab	8 weeks
Ponraj 2023 [17]	2	F	70	Eculizumab	12 weeks
		F	81	Eculizumab	Not achieved
Meseha 2024 [16]	1	M	77	Eculizumab	Not achieved
Attucci 2024 [18]	2	M	75	Eculizumab	8 weeks
		F	72	Eculizumab	7 weeks
Ceglédi 2024 [19]	1	F	47	TPE alone	3 weeks
Lopes 2025 [20]	1	F	73	Eculizumab	8 weeks

Abbreviation: TPE, therapeutic plasma exchange.

TMA is characterized by platelet thrombus formation in microarterioles or capillaries, resulting in MAHA and thrombocytopenia [21]. Key clinical manifestations encompass AKI and central nervous system involvement. The growing interest in TMA stems from its heterogeneous etiologies and substantial mortality risk. In our cases, rapid hemoglobin decline, schizocytes on peripheral blood smear, elevated LDH, and a negative DAT confirmed MAHA, while severe thrombocytopenia and AKI suggested hemolytic uremic syndrome (HUS), a TMA subtype.

Primary TMA in adults arises from diverse etiologies, including thrombotic thrombocytopenic purpura (TTP), Shiga toxin‐mediated HUS (ST‐HUS), complement‐mediated TMA (CM‐TMA), and drug‐induced TMA (DITMA) [22]. DITMA is a potentially life‐threatening disease [23], often triggered by medications such as chemotherapeutics. In our cases, the absence of gastrointestinal infection, normal ADAMTS13 activity, and symptoms post‐CFZ chemotherapy suggested DITMA. DITMA develops when drugs induce platelet thrombus formation in microvessels, leading to TMA via mechanisms including direct endothelial injury, immune‐mediated responses, and coagulation pathway dysregulation.

Carfilzomib, a proteasome inhibitor used for relapsed/refractory MM (typically as second‐line therapy), differs mechanistically from bortezomib: while both inhibit the proteasome, CFZ binds irreversibly, whereas bortezomib binds reversibly. CFZ can cause TMA with a cumulative effect dose that mainly affects kidney endothelial cells [23]. Joseph et al. [12] synthesized 37 CFZ‐TMA cases, reporting a median latency of 3.5 months from first CFZ exposure to TMA onset. Over half of patients had predisposing factors, with influenza infection being the most common trigger (mirroring Case 2 in our series). All patients experienced AKI, with 84% in stage 3 according to KDIGO criteria. ADAMTS13 levels were normal in 76% of patients, and complement activity was normal in 49%, with no pathogenic variants identified.

The pathogenesis of CFZ‐TMA involves multiple factors. While mutations in genes related to the VEGF pathway and the complement alternative pathway are considered as potential causes, emerging evidence refines this understanding. CFZ‐mediated inhibition of the VEGF pathway may attenuate the protective effect of renal microvasculature against complement‐mediated injury, thereby elevating the risk of renal microvascular injury [24, 25]. Moscvin et al. reported that 7 out of 10 cases of CFZ‐TMA had deletions in the *CFHR3‐CFHR5* region, implicating alternative complement pathway dysfunction as a potential risk factor [26]. However, a recent review highlighted that complement genetic pathogenic mutations are rare in DITMA, occurring in only 2 of 122 cases (1.6%) [23], underscoring that non‐genetic triggers predominate in most cases. Additionally, a study by Gavriilaki et al. [27] found elevated complement activation markers (e.g., C5b‐9) in myeloma patients developing CFZ‐TMA, supporting complement pathway involvement. Our cases align with these findings: normal ADAMTS13, no gastrointestinal triggers, and TMA onset shortly after CFZ initiation Genetic testing for complement pathway abnormalities, including *CFHR3‐CFHR5* deletions, could provide further insights into the susceptibility of these patients to TMA.

Although CFZ was withdrawn and plasma exchange initiated soon after TMA was suspected, renal function did not significantly improve, and MAHA persisted in both patients. Therapeutic plasma exchange, the most recommended front‐line regimen, was performed in 51% of patients, and corticosteroid therapy in 38%. However, these treatments did not significantly impact early survival, TMA remission, or renal function recovery rates. The early mortality rate was 19%, and late‐stage chronic renal failure sequelae occurred in 38%. Given the importance of complement activation in the pathogenesis, early identification and intervention with complement inhibitors like eculizumab might be crucial in managing such cases.

Many case reports highlight eculizumab's effectiveness in treating DI‐TMA and reducing adverse outcomes [17, 28, 29, 30]. Eculizumab, a monoclonal antibody targeting complement C5, inhibits C5b‐9 formation and has shown efficacy in preserving renal function. Despite initiating eculizumab 14 days post‐onset, the patient achieved favorable outcomes, suggesting benefits even with late administration. Zafar et al. [31] reviewed 69 cases of drug‐induced TMA (including 11 CFZ cases) and proposed eculizumab as a potential treatment for severe or refractory DI‐TMA after initial treatment failure. Through our literature review, we found that since 2017, eculizumab has emerged as a first‐line therapeutic option for CFZ‐TMA, with a considerable number of patients achieving clinical remission after switching to eculizumab following TPE failure. Joseph et al. [12] summarized clinical data from 37 CFZ‐TMA patients in the French Reference Center for TMA, revealing that 50% of these patients received eculizumab. At final follow‐up, 62% of all enrolled patients achieved normalization of renal function. Fang et al. [32] collected and analyzed case data from 66 CFZ‐TMA patients, identifying that 24.2% were treated with eculizumab. Among all patients, 71.2% achieved clinical symptom resolution, which is consistent with the findings of the present study.

In future cases, special attention should be given to patients with acute viral infections or previous platelet count or creatinine fluctuations. Close monitoring during diagnosis and treatment is essential. CFZ‐TMA may involve abnormal complement activation, making complement inhibitors crucial interventions. Early treatment may significantly improve prognosis.

## Author Contributions

Zi Wang and Junling Zhuang conceived the study idea. Zi Wang and Hua Zheng collected the data, and drafted the manuscript. Wei Wang and Xiaoxiao Shi collected the data and performed the data analysis. Yunpeng Wei and Hua Zheng drew the figures. Chao Li and Zi Wang revised the article. Junling Zhuang supervised the entire process.

## Ethics Statement

Ethical approval was not required for this case report in accordance with local legislation and institutional requirements.

## Consent

Written informed consent was obtained from the patient for publication of this case report.

## Conflicts of Interest

The authors declare no conflicts of interest.

## Data Availability

The data that support the findings of this study are available on request from the corresponding author.
